# Visceral Fat Thickness, Serum Adiponectin, and Metabolic Syndrome in Patients with Colorectal Adenomas

**DOI:** 10.3390/jpm14091008

**Published:** 2024-09-22

**Authors:** Dimitrije Damjanov, Tijana Ičin, Željka Savić, Nebojša Janjić, Stanislava Nikolić, Olgica Latinović Bošnjak, Žarko Krnetić, Vladimir Vračarić, Božidar Dejanović, Nadica Kovačević

**Affiliations:** 1 Faculty of Medicine, University of Novi Sad, 21137 Novi Sad, Serbia; tijana.icin@mf.uns.ac.rs (T.I.); zeljka.savic@mf.uns.ac.rs (Ž.S.); nebojsa.janjic@mf.uns.ac.rs (N.J.); stanislava.nikolic@mf.uns.ac.rs (S.N.); bozidar.dejanovic@mf.uns.ac.rs (B.D.); nadica.kovacevic@mf.uns.ac.rs (N.K.); 2 Clinic for Gastroenterology and Hepatology, University Clinical Center of Vojvodina, 21137 Novi Sad, Serbia; olgica.latinovic@gmail.com (O.L.B.); zarkokrnetic@yahoo.com (Ž.K.); vracaricv@gmail.com (V.V.); 3 Clinic for Endocrinology, Diabetes and Metabolic Diseases, University Clinical Center of Vojvodina, 21137 Novi Sad, Serbia; 4 Center for Laboratory Medicine, University Clinical Center of Vojvodina, 21137 Novi Sad, Serbia; 5 Clinic for Infectious Diseases, University Clinical Center of Vojvodina, 21137 Novi Sad, Serbia

**Keywords:** visceral fat, adiponectin, metabolic syndrome, colorectal adenoma, colorectal cancer, ultrasound, obesity

## Abstract

Background/Objectives: Most cases of colorectal cancer (CRC) arise from adenomatous polyps. Identifying risk factors for colorectal adenoma (CRA) is critical for CRC prevention. Emerging evidence suggests a link between metabolic syndrome (MetS) and an elevated risk of CRA and CRC, potentially mediated by visceral obesity and adiponectin (APN). We aimed to evaluate the association between different markers of visceral obesity, serum APN, MetS, and the presence of CRA. Methods: A cross-sectional study was conducted at the University Clinical Center of Vojvodina, involving 120 patients, aged 40–75 years, who underwent colonoscopy between January 2022 and January 2023. Sixty patients with CRA were compared to 60 controls with normal colonoscopy findings. Visceral fat thickness (VFT) was measured using ultrasound (US), and bioelectrical impedance analysis (BIA) was used to assess visceral fat area (VFA). Serum APN levels, anthropometric measures, and MetS components were also evaluated. Results: Patients with CRA had significantly higher VFT measured by US (p < 0.05), but no significant differences were found in VFA measured by BIA, waist circumference (WC), or waist-to-hip ratio (WHR). MetS was significantly more prevalent in the CRA group (55% vs. 31.6%, p < 0.05), and logistic regression confirmed MetS as a significant predictor of CRA presence (OR = 2.6). Serum APN levels were inversely correlated with visceral fat measurements and MetS (p < 0.01), but no significant difference in APN levels was observed between patients with and without CRA. Conclusions: This study highlights the importance of VFT measured by US and the presence of MetS as significant factors associated with CRA.

## 1. Introduction

Colorectal cancer (CRC) is the third most prevalent malignancy globally and the second leading cause of cancer-related deaths [1]. Most CRC cases originate from polyps, with the adenoma–carcinoma sequence accounting for three-quarters of these transitions [2]. Therefore, identifying risk factors for colorectal adenoma (CRA) is crucial for effective screening, risk modification, and CRC prevention [3]. Risk factors associated with colorectal neoplasms include a positive family history of colorectal cancer or polyps, inflammatory bowel diseases (IBD), cigarette smoking, insufficient physical activity, and obesity [4].

Literature data indicate that patients with metabolic syndrome (MetS) have an elevated risk of CRA and CRC [5]. The occurrence of cancer in these patients is mainly explained by the presence of insulin resistance and the influence of insulin-like growth factor 1 (IGF-1), with additional factors such as free fatty acids, aromatase, and adipokines [6].

Obesity plays a crucial role in initiating insulin resistance, leading to systemic diseases and organ-specific disorders [7]. The most metabolically active endocrine organ is adipose tissue, which is the source of adiponectin (APN), interleukin (IL)-6, and tumour necrosis factor-alpha (TNF-α). Circulating APN inversely correlates with the amount of visceral fat tissue, and lower APN concentrations are observed in MetS, type 2 diabetes mellitus, cardiovascular diseases, and certain malignancies [8].

For quantitative assessment of visceral fat tissue, CT and MRI are considered the gold standard methods. However, both are expensive and may be contraindicated for certain individuals; additionally, CT involves radiation exposure, and MRI is not widely available [9]. Methods explored in this study were ultrasound (US), bioelectrical impedance analysis (BIA), and anthropometric measurements. US is safe, accessible, and quick to perform. US measurement of visceral fat thickness (VFT) from the posterior edge of the rectus abdominis muscle to the anterior wall of the aorta correlates well with visceral fat measured by CT. BIA is an accessible and safe method for measuring body composition, but remains insufficiently accurate for assessing visceral fat tissue. Anthropometric measurements (waist circumference (WC), waist-to-hip ratio (WHR), BMI) are the least precise for assessing visceral fat tissue [9].

The objectives of this study were to determine the difference in the amount of visceral fat tissue between patients with and without CRA; assess the difference in serum APN levels and the presence of MetS between patients with and without CRA; and evaluate the association between the size, number, location, and histopathological characteristics of CRA and MetS and the amount of visceral fat tissue.

## 2. Materials and Methods

We conducted a cross-sectional study at the Clinic for Gastroenterology and Hepatology, University Clinical Center of Vojvodina. The study was approved by the institutional ethics committee. Patients, aged 40–75 years, who had undergone total colonoscopy between January 2022 and January 2023 were invited for an informative interview. The study group consisted of 60 consecutive patients who had been endoscopically and histologically diagnosed with CRA. The control group comprised 60 consecutive patients with normal findings on colonoscopy. All patients signed the informed consent. All patients were Caucasian. Exclusion criteria were insufficiently cleansed colon, CRC, IBD, colon resection, insulin treatment, hyperplastic polyp, and pregnancy.

Colonoscopies were performed by experienced gastroenterologists. Data routinely documented during colonoscopy and from histopathological reports were collected from medical records, including polyp size (≤5 mm, 6–9 mm and ≥10 mm); polyp morphology (sessile, pedunculated, or flat); number of polyps (1, 2, or ≥3); polyp distribution (polyps found in the cecum, ascending colon, and transverse colon were classified as proximal colon, while those located in the splenic flexure, descending colon, sigmoid colon, and rectum were classified as distal colon). Adenomas were considered advanced if they were ≥10 mm in size, had high-grade dysplasia, and/or contained a ≥25% villous component [10].

Participants were interviewed and asked about age and sex; cigarette smoking (participants were categorised based on their lifetime consumption of 100 cigarettes and current smoking status into non-smokers, former smokers, and smokers); alcohol consumption (consumption of more than two standard alcoholic drinks per day was considered significant; one standard alcoholic drink was defined as one 330 mL glass of beer (5% alcohol), 40 mL of a spirit (40% alcohol), or 140 mL of wine (12% alcohol)); family history (presence of CRC in first-degree relatives); use of ASA and NSAIDs (daily use of these medications for the past year was considered significant); physical activity (participants who engaged in more than 150 min of moderate-intensity physical activity per week or more than 75 min of vigorous-intensity physical activity per week were classified as physically active); arterial hypertension, diabetes, low HDL cholesterol (HDL-C), and high triglycerides; medications in use.

Blood samples were collected from patients in the morning hours after a 12 h fasting period. The following parameters were assessed: glucose, triglycerides, HDL-C, and APN concentrations. Serum glucose was measured using the standard enzyme-specific GOD-PAP method. Triglycerides were measured in serum using a standard enzymatic procedure with the enzyme lipase. HDL-C was determined in serum using a direct enzymatic method for quantitative HDL-C measurement. Serum APN (samples stored at −80 °C for two months) was measured using the ELISA method.

Patients had their height and weight measured, after which their BMI was calculated. Blood pressure was measured after a ten-minute rest using a sphygmomanometer and stethoscope. Waist and hip circumference were measured according to WHO recommendations. Subsequently, the waist-to-hip ratio (WHR) was calculated.

Visceral fat thickness (VFT) was measured using US with a multifrequency convex probe (3.0–5.0 MHz). Measurements were taken with the subject in the dorsal decubitus position following a 12 h fasting period. The probe was placed transversely 1 cm from the umbilicus along the xipho-pubic line, ensuring no pressure was applied to the abdomen to avoid altering VFT. The boundaries of visceral fat were determined by the linea alba and the anterior wall of the aorta during patient exhalation. All US examinations were conducted by a specialist in internal medicine specifically trained in VFT measurement.

BIA was conducted using the InBody 770 device, which calculated the visceral fat area (VFA).

Diagnosis of MetS was based on the latest criteria from leading associations, with participants meeting at least three of the following five criteria: 1. increased WC: men ≥ 94 cm, women ≥ 80 cm; 2. hypertriglyceridemia (or taking medication to lower triglycerides): ≥1.7 mmol/L; 3. low HDL-C (or taking medication to increase HDL-C): men < 1.0 mmol/L, women < 1.3 mmol/L; 4. elevated blood pressure (or taking antihypertensive medication): systolic ≥ 130 mmHg and/or diastolic ≥ 85 mmHg; 5. fasting hyperglycaemia (or taking antihyperglycaemic medication): ≥5.6 mmol/L [11].

### Statistical Analysis

Data entry and analysis were conducted using IBM SPSS 22.0. Descriptive statistics were presented as means and standard deviations for continuous variables, and as frequencies and percentages for categorical variables. Depending on the type of variable, comparisons between groups were performed using *t*-tests, ANOVA, or the Mann–Whitney U test. Pearson and Spearman correlation coefficients were used to assess correlations between variables. Statistical significance was set at 95% (*p* < 0.05) and 99% (*p* < 0.01) confidence levels for the applied tests.

## 3. Results

### 3.1. Demographic Characteristics, Protective and Risk Factors

Among CRA patients, 46.7% were male, while 40% of the control group were male, showing no statistically significant difference in gender distribution (Table 1).

The average age in the CRA group was 60.10 ± 7.99 years, while in the control group, it was 56.75 ± 9.26 years. The difference in average age between the two groups was statistically significant (Table 1).

There was no statistically significant association between the frequency of protective factors and risk factors and group affiliation (Table 2).

### 3.2. Polyp Characteristics

Table 3 presents the characteristics of polyps in the group of patients with CRA. In two thirds of the patients, the polyps were ≥10 mm. In 55% of the cases, the polyps were pedunculated. The majority of patients had a single polyp. In more than three quarters of the cases, the polyps were localized in the distal colon. Advanced polyps were recorded in 70% of the patients.

### 3.3. Markers of Visceral Obesity

Correlation analysis results demonstrate that all three methods of measuring visceral obesity positively correlate with each other, indicating that individuals with increased WC also tend to have increased VFA measured by BIA and increased VFT measured by US (Table 4).

Table 5 shows average values of BMI, WC, WHR, VFA, and VFT. The significant difference in markers of visceral obesity between the two groups was present only in case of VFT measured by US, in favour of higher values in patients with CRA.

There were no significant associations between the markers of visceral obesity and any of the examined CRA characteristics.

### 3.4. Metabolic Syndrome

MetS was present in 55% of patients with CRA, compared to 31.6% in the control group. There was a statistically significant association between the presence of MetS and CRA (χ^2^ = 6.652, df = 1, rφ = −0.235, *p* < 0.05).

To test if the presence of MetS predicts the presence of CRA in patients, binary logistic regression was conducted (Table 6). The final model was statistically significant, indicating that MetS significantly predicts the presence of CRA (OR = 2.6).

Further logistic regression including additional factors (sex, age, smoking status, alcohol consumption, family history of CRC, ASA and/or NSAID use, and physical activity) (Table 7) indicated that MetS significantly predicts the presence of CRA (χ^2^ = 17.750, df = 9, *p* < 0.05, Nagelkerke R Square = 0.183). The model explained 18.3% of the variance in CRA presence and correctly classified 62.5% of patients. However, MetS remained the only statistically significant predictor for CRA presence among the included parameters. The model’s sensitivity was 63.3%, and specificity was 61.7%.

There were no significant associations between MetS and any of the examined CRA characteristics.

### 3.5. Serum Adiponectin

A significant difference in serum APN levels was found between patients with MetS and those without. Patients with MetS had significantly lower serum APN levels (Table 8).

Spearman’s rank correlation coefficient showed a significant negative association of moderate to high intensity between APN levels and the amount of visceral fat tissue for all three measurement techniques (WC, VFA by BIA and VFT by US). This indicates that individuals with lower APN levels tend to have higher WC, VFA and VFT (Table 9).

There was no statistically significant difference in serum APN levels between patients with and without CRA (Table 10).

## 4. Discussion

Our study aimed to compare different markers of visceral obesity, prevalence of MetS, and serum APN levels between patients with CRA and those with normal colonoscopy findings.

In our study, no statistically significant difference in BMI was found between the group of patients with CRA and the control group. Similarly, a meta-analysis by Keum et al., which explored the relationship between visceral obesity and CRA, concluded that BMI is not an independent risk factor for adenomas [12].

Studies have shown that an increased WC raises the risk of CRA by 43% [13]. In a Chinese study examining the association between fat distribution and MetS with CRA, multivariate logistic analysis showed that increased WHR carries a risk for adenoma development [14]. In our study, the average WC in the subgroup of male patients with CRA did not differ significantly from the average WC of men in the control group. No significant differences were found between these two groups in females either. Similarly, WHR did not differ significantly between patients with CRA and the control group. The meta-analysis investigating the relationship between visceral obesity and CRA suggested that WC is not an independent risk factor for adenomas but gains significance in the presence of a larger amount of visceral fat measured by CT [12].

In our study, VFA values measured by BIA did not differ significantly between patients with CRA and the control group. Review of the available literature has revealed a lack of adequate or at least current data on the relationship between VFA measured by BIA and the presence of CRA.

Our study shows VFT values measured by US are statistically significantly higher in patients with CRA compared to the control group. Based on a review of the available literature, this research is the first to examine the association between CRA and VFT measured by US. US is an accessible method, simple to perform, and correlates well with CT in assessing visceral obesity, which is the gold standard for estimating the amount of visceral fat tissue [15,16]. An analysis of six observational studies examining the relationship between VFA measured by CT and the presence of CRA showed a dose-dependent correlation [12].

VFT measured by US was the only marker of visceral obesity that statistically significantly differed between patients with CRA and the control group, with higher values in patients with CRA. In contrast, the results for WC, WHR, and VFA measured by the BIA method did not show significant differences between the two groups. Considering our results and the fact that USG is a more precise method for assessing intra-abdominal fat tissue, it can be said that patients with CRA indeed had more visceral fat tissue than the control group. The absence of differences in WC, WHR, and VFA measured by the BIA method between these two groups was likely due to the lower precision of anthropometric measurements and the BIA method in differentiating visceral obesity.

Our research did not find an association between visceral obesity and the dimensions, number, distribution, or advancement of CRA. This lack of association was also found in a study where VFA was measured by CT [17]. Nagata and colleagues quantified visceral fat tissue using CT and reported a higher frequency of advanced adenomas, multiple adenomas, and adenomas in proximal locations in subjects with higher VFA [18].

In our study, MetS was significantly more prevalent in patients with CRA, aligning with the findings of a meta-analysis that explored the association between MetS and colorectal neoplasms [19]. Results of our logistic regression including additional factors (sex, age, smoking, alcohol consumption, family history, ASA and other NSAIDs, and physical activity) showed that MetS remained the only statistically significant predictor for CRA presence among the included parameters. Based on our study results, individuals with MetS are 2.6 times more likely to have CRA. This risk is higher (OR 1.39) than in a meta-analysis published in 2021, although it is important to note that different criteria for MetS and varied populations across studies in the meta-analysis could have influenced the results [13].

Our findings do not indicate a higher incidence of advanced adenomas, larger adenomas, multiple adenomas, or adenomas in multiple segments of the colon in patients with MetS compared to the control group. This contrasts with other studies that have shown MetS correlates significantly with adenomas in multiple locations [20], multiple adenomas [21], larger adenomas [22], and advanced adenomas [23]. In the Portuguese study, subjects with MetS had more frequent multiple adenomas, but there were no differences in the size, distribution, or advanced nature of adenomas compared to those without MetS [24]. An Italian study found MetS significantly associated with the presence of both polypoid and non-polypoid changes in the colon, with no differences in their size and number between subjects with MetS and the control group [5].

As expected, our study results showed significantly lower serum APN levels in patients with MetS compared to those who had not met enough criteria for this syndrome. The meta-analysis investigating the potential use of circulating APN levels as a biomarker for MetS indicated that hypoadiponectinemia predicted the incidence increase in MetS linearly and dose-dependently [25].

Our study found a statistically significant negative correlation, moderate to high intensity, between APN levels and the amount of visceral fat tissue, as measured by all three techniques. This means that individuals with low APN levels had a larger WC, higher VFA measured by the BIA method, and higher VFT measured by US. It is well known that APN levels decrease with obesity, especially in the abdominal type [26,27].

In our study, no significant difference was found in serum APN levels between patients with CRA and those without CRA. This is contrary to a meta-analysis that showed low APN levels associated with CRC and CRA, although the analysis itself was highly heterogeneous [28]. APN is the predominant adipokine but not the only one that can explain the link between visceral obesity and CRA.

This study is the first to investigate visceral obesity, MetS, and APN levels in patients with CRAs compared to patients with normal colonoscopy findings in our region. Generally, there is limited literature on the association between various markers of visceral obesity and CRA.

Although there are some weaknesses in our study (relatively small sample size and cross-sectional design), it also has many strengths: we considered various confounding factors, including demographics, lifestyle habits, family history, and the use of NSAIDs. The study is the first to explore the relationship between CRA and visceral fat measured by US.

## 5. Conclusions

The findings of this study highlight the significance of MetS and visceral obesity in patients with CRA. However, they also underscore the need for further research involving larger sample sizes, additional methods for assessing visceral obesity, and the measurement of other adipokines to improve CRC risk management.

## Figures and Tables

**Table 1:** Demographic characteristics in CRA and control group.

	CRA	Control Group	Test	df	*p*
**Gender** [*n* (%)]					
Male	28 (46.7)	24 (40.0)	χ^2^ = 0.543	1	>0.05
Female	32 (53.3)	36 (60.0)			
**Age** (years) (mean ± SD)	60.10 ± 7.99	56.75 ± 9.26	t = 2.12	118	**<0.05**

**Table 2:** Protective and risk factors in CRA and control group.

	CRA [*n* (%)]	Control [*n* (%)]	χ^2^	df	*p*
**Smoking status**					
Smokers	20 (33.3)	10 (16.7)			
Ex-smokers	18 (30.0)	18 (30.0)			
Never smokers	22 (36.7)	32 (53.3)	5.185	2	>0.05
**Alcohol consumption**					
Yes	6 (10.0)	4 (6.7)			
No	54 (90.0)	56 (93.3)	0.436	1	>0.05
**Family history of CRC**					
Yes	15 (25.0)	19 (31.7)			
No	45 (75.0)	41 (68.3)	0.657	1	>0.05
**ASA and/or NSAIL use**					
Yes	12 (20.0)	11 (18.3)			
No	48 (80.0)	49 (81.7)	0.054	1	>0.05
**Physical activity**					
Yes	52 (86.7)	48 (80.0)			
No	8 (13.3)	12 (20.0)	0.960	1	>0.05

**Table 3:** Polyp characteristics.

	N (%)
**Size**	
≤5 mm	3 (5.0)
6–9 mm	18 (30.0)
≥10 mm	39 (65.0)
**Morphology**	
Sessile	27 (45.0)
Pedunculated	33 (55.0)
**Number**	
1	44 (73.3)
2	15 (25.0)
≥3	1 (1.7)
**Localisation**	
Proximal colon	9 (15.0)
Distal colon	46 (76.7)
Both	5 (8.3)
**Advanced adenoma**	
Yes	42 (70.0)
No	18 (30.0)

**Table 4:** Association between different methods of measuring visceral obesity.

	WC	BIA	US
**WC**			
**BIA**	0.613 **		
**US**	0.868 **	0.586 **	

** *p* < 0.01.

**Table 5:** Markers of visceral obesity and group comparisons.

	CRA (Mean ± SD)	Control (Mean ± SD)	t	df	*p*
**BMI** (kg/m^2^)	28.78 ± 4.82	27.39 ± 4.75	1.585	118	>0.05
**WC** (cm)	92.28 ± 13.13	89.25 ± 13.16	1.264	118	>0.05
**WHR**	0.88 ± 0.09	0.86 ± 0.08	0.933	118	>0.05
**VFA** (cm^2^)	140.88 ± 55.58	123.32 ± 49.87	1.821	118	>0.05
**VFT** (mm)	56.03 ± 26.2	46.60 ± 22.89	2.097	118	**<0.05**

**Table 6:** Predicting CRA with MetS.

Predictor	B	SE	Wald	df	*p*	Exp(B)	95% CI
MetS	0.970	0.380	6.515	1	**0.011**	2.637	1.252–5.554

**Table 7:** The results of the binary logistic regression in predicting the presence of CRA.

Predictor	B	SE	Wald	df	*p*	Exp(B)	95% CI
MetS	0.943	0.456	4.272	1	**0.039**	2.568	1.050–6.280
Gender (1)	−0.404	0.445	0.823	1	0.364	0.668	0.279–1.597
Age	−0.034	0.027	1.555	1	0.212	0.967	0.917–1.019
Smoking			6.051	2	0.051		
Smoking (1)	0.257	0.473	0.295	1	0.587	1.293	0.511–3.269
Smoking (2)	−1.015	0.568	3.196	1	0.074	0.362	0.119–1.103
Alcohol (1)	−0.009	0.791	0.000	1	0.991	0.991	0.211–4.667
Family history (1)	−0.280	0.455	0.379	1	0.538	0.756	0.310–1.844
ASA (1)	−0.441	0.549	0.643	1	0.423	0.644	0.219–1.889
Physical activity (1)	0.718	0.580	1.532	1	0.216	2.051	0.658–6.394

**Table 8:** Serum adiponectin and the presence of MetS.

	Group	Mean Rank	Sum of Ranks	U	Z	*p*
**Adiponectin** (µg/mL)	MetS—yes	45.10	2345.00	967.00	−4.242	**<0.01**
	MetS—no	72.28	4915.00			

**Table 9:** Serum adiponectin and markers of visceral obesity.

	APN	WC	VFA	VFT
**APN**				
**WC**	−0.588 **			
**VFA**	−0.289 **	0.625 **		
**VFT**	−0.589 **	0.880 **	0.627 **	

** *p* < 0.01.

**Table 10:** Serum adiponectin in CRA and control group.

	Group	Mean Rank	Sum of Ranks	U	Z	*p*
**Adiponectin** (µg/mL)	CRA	55.18	3310.50	−1.986	−1.677	>0.05
	Control	65.83	3949.50			

## Data Availability

The data that support the findings of this study are available from the corresponding author upon reasonable request.
